# Supplementary material for: Effects of mechanical insufflation-exsufflation on ventilator-free days in intensive care unit subjects with sputum retention; a randomized clinical trial
Source: PLoS One. 2024 May 2;19(5):e0302239. doi: 10.1371/journal.pone.0302239 (PMC11065296; doi:10.1371/journal.pone.0302239)
Supplement: S1 File — (PDF) [file pone.0302239.s002.pdf]

Clinical Research

The Efficacy of Mechanical Airway Clearance Assistance in Patients with Mechanical Ventilation Admitted to the Intensive Care Unit (ICU)

Research Implementation Plan

Research Leader:

Kensuke Nakamura, Department of Emergency and Critical Care Medicine, Hitachi General Hospital

Research Team Members:

Tomohiro Sonoo, Department of Emergency and Critical Care Medicine, Hitachi General Hospital

Makiko Tomioka, Department of Nursing in Emergency and Critical Care Center(3-2・3-3 ward), Hitachi General Hospital

Yurika Yoshikawa, Department of Nursing in Emergency and Critical Care Center(3-2・3-3 ward), Hitachi General Hospital

Date of Creation:

Plan Draft September 13, 2017

Table of Contents:

0. Overview

1. Research Background

2. Research Objectives

3. Patients and Eligibility Criteria

4. Research Methods

5. Observation and Examination Items

6. Expected Benefits and Disadvantages (Side Effects)

7. Endpoints

8. Handling of Adverse Events

9. Discontinuation Criteria for Individual Subjects

10. Research Implementation Period

11. Target Number of Cases and Rationale, as well as Statistical Analysis Methods

12. Considerations for Human Rights of Subjects and Methods for Protecting Personal Information

13. Response and Compensation for Health Damage to Subjects

14. Expenses Incurred by Subjects

15. Record Keeping and Publication of Research Results

16. Research Funding and Conflicts of Interest

17. Research Implementation Structure

18. References and Literature List

0. Overview

(1) Purpose

To clarify the effectiveness of Mechanical Insufflator-Exsufflator (MI-E) in patients with mechanical ventilation support admitted to the ICU.

(2) Subjects

60 patients with mechanical ventilation support admitted to the ICU during the research period.

(3) Research Methods

Selecting 60 patients from those admitted to the ICU who required frequent suctioning among those needing mechanical ventilation management. Divide them into two groups: cases where only traditional pulmonary care is provided (non-intervention group) - 30 cases, and cases where MI-E is used in addition to traditional pulmonary care (intervention group) - 30 cases, to prospectively verify the effectiveness of MI-E. The investigation items include the number of days of ventilator support, re-intubation rate, tracheostomy rate, P/F ratio, CRP, PCT, and days in ICU, and they will be compared between the two groups.

(4) Evaluation Items (Endpoints)

Primary outcome:

number of ventilator-free days

Secondary outcomes:

ICU length of stay, mortality rate, tracheostomy rate, P/F ratio, CRP, PCT

(5) Planned Enrollment: 60 patients

(6) Research Period: From approval by the ethics committee to September 30, 2019

1. Research Background

Mechanical Insufflator-Exsufflator (MI-E) is a device used to improve airway clearance by applying positive and negative pressure to the airway and lungs through a mask for non-intubated patients or by connecting it to an endotracheal tube for intubated patients. It is known to be effective in preventing atelectasis, maintaining lung and chest wall mobility, and assisting deep breathing. Currently, the use of MI-E is recommended in guidelines for patients with neuromuscular diseases and spinal cord injuries.[1)2)]

ICU patients often experience reduced cough function due to conditions like altered consciousness, sedation, pain, and immobility, leading to decreased airway clearance. Reduced airway clearance can result in dyspnea, respiratory muscle fatigue, unstable breathing patterns, and prolonged mechanical ventilation. These factors can lead to extended ICU stays, reduced activities of daily living (ADL) due to muscle weakness, and exacerbation of delirium, significantly impacting patient function and prognosis.

The use of MI-E in acute care patients has started to gain attention, with case reports and preliminary studies suggesting its effectiveness.[3)] However, there is limited reporting and research on the use and effectiveness of MI-E in intubated patients. Our ward has been using Confort Cough for about a year now, both for non-intubated and intubated patients. In many cases, we have found it to be effective in aiding mucus drainage in addition to traditional

pulmonary care. Therefore, we want to focus on intubated patients with mechanical ventilation support and investigate the effectiveness and safety of MI-E in the acute care setting.

2. Research Objectives

To clarify the effectiveness of Mechanical Insufflator-Exsufflator (MI-E) in patients with mechanical ventilation support admitted to the ICU.

3. Subjects and Eligibility Criteria

The study will target patients admitted to the ICU where comprehensive management is provided by the Intensive Care Unit. The following selection criteria and exclusion criteria will be applied:

(1) Selection Criteria:

Patients aged 18 years or older at the time of obtaining consent

Patients who have been on mechanical ventilation support for 24 hours or longer and are predicted to require mechanical ventilation support for 48 hours or more

Patients with a significant amount of sputum (requiring suctioning approximately once per

hour) and for whom the physician deems pulmonary physiotherapy necessary

Patients who have received sufficient explanation and have provided written consent based on their own free will after adequate understanding for participation in this study

(2) Exclusion Criteria:

Patients whom the Principal Investigator deems unsuitable as subjects

4. Research Methods

(1) Type and Design of the Research

Prospective, non-randomized, non-blinded intervention trial

(2) Research Outline

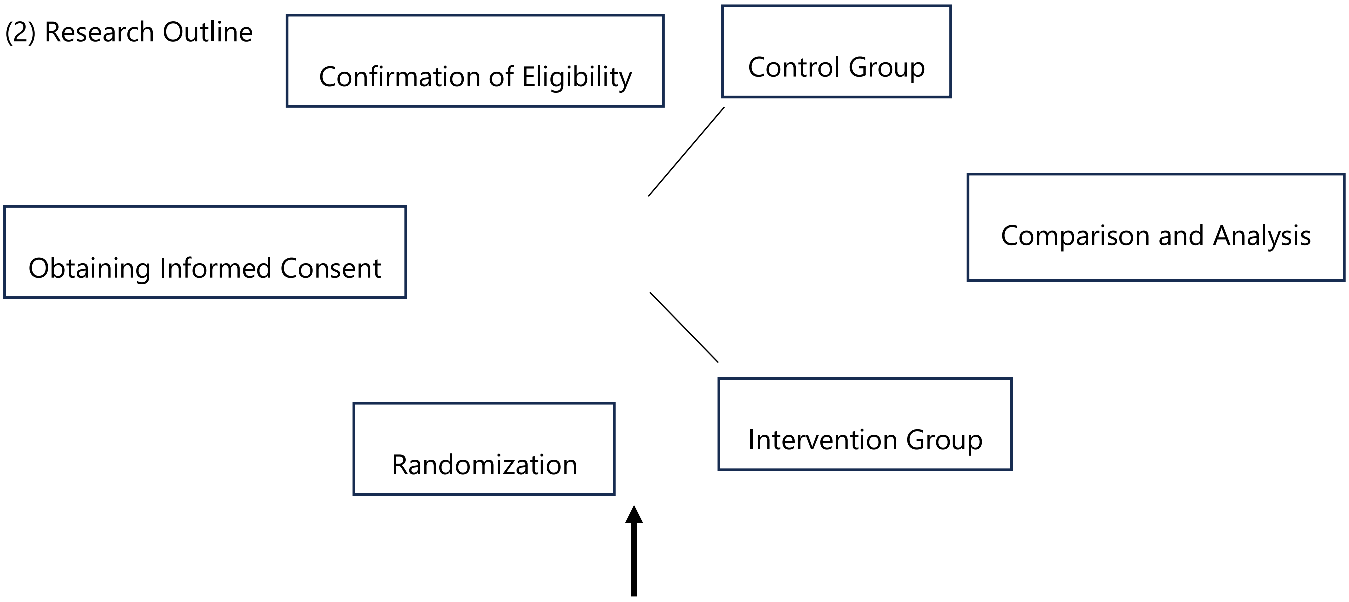

Hospitalization	Commencement	Duration of	End of Observation

(3) Provisions for Concomitant Therapy

None

(4) Methods for Case Registration and Allocation

Random allocation using a randomization table

(5) Expected Duration of Subject Participation

The enrollment period is from approval by the ethics committee until June 30, 2019.

All patients meeting the inclusion criteria.

5. Observation and Examination Items

①Patient data: Age, gender, diagnosis

②APACHE II score

③Days with mechanical ventilation support

④Tracheostomy rate

⑤Re-intubation rate

⑥Blood sample data (CPR, PCT)

⑦Blood gas data

⑧the ICU length of stay

6. Expected Benefits and Disadvantages (Side Effects)

(1) Expected Benefits

Potential reduction in the ICU length of stay and days with mechanical ventilation support.

Potential contribution to the advancement of medical knowledge.

(2) Expected Disadvantages (Side Effects)

There is a possibility of air leaks such as pneumothorax or subcutaneous emphysema due to pressure on the lungs. However, it is believed that air leaks will not occur with the positive pressure of 40 cmH_2O used in this study.

7.Endpoints

(1) Primary Outcome:

number of ventilator-free days

(2) Secondary Outcomes:

ICU length of stay, mortality rate, tracheostomy rate, P/F ratio, CRP, PCT

8. Handling of Adverse Events

Adverse events will be documented in case reports.

9. Discontinuation Criteria for Individual Subjects

(1) Withdrawal of consent by the patient or their surrogate

(2) Request for changes or discontinuation of treatment by the patient or their surrogate

(3) Occurrence of adverse events (deterioration of the underlying condition, worsening of complications, development of new conditions, etc.) that the treating physician deems inappropriate to continue the trial

10. Research Implementation Period

From approval by the ethics committee until September 30, 2019

11. Target Number of Cases and Statistical Analysis Methods

(1) Target Number of Cases

60 cases are planned for enrollment.

(2) Statistical Analysis Methods

Comparison of each evaluation item for patient severity and test results will be performed. To examine the comparison between the intervention group and the non-intervention group, paired t-tests will be used for analysis.

12. Consideration for Human Rights of Subjects and Protection of Personal Information

All personnel involved in this research will conduct the study in accordance with the "Declaration of Helsinki (2008 Revision)" and the "Ethical Guidelines for Clinical Research (Revised on July 31, 2008)."

When handling samples and related materials for research purposes, they will be managed with unrelated numbers to individual information and with due consideration for the

confidentiality of subjects. When publishing the results of the research, information that could identify the subjects will not be included. In addition, the samples and materials obtained in the research will not be used for purposes other than the research.

13. Response and Compensation for Health Damage to Subjects

Not applicable.

14. Expenses Incurred by Subjects

There are no expenses incurred by subjects.

15. Record Keeping and Publication of Research Results

The research leader will appropriately store important documents related to the research within the facility and keep them for a period of 10 years after the completion or termination of the research. Afterward, they will be discarded with attention to personal information. The research results will be published by the research personnel through presentations at relevant scientific conferences, etc.

16. Research Funding and Conflicts of Interest

Not applicable.

17. Research Implementation Structure

This research will be conducted with the following structure:

[Research Members]

○Kensuke Nakamura, Director of Department of Emergency and Critical Care Medicine, Hitachi General Hospital

Tomohiro Sonoo, Department of Emergency and Critical Care Medicine, Hitachi General Hospital

Makiko Tomioka, Department of Nursing in Emergency and Critical Care Center(3-2・3-3 ward), Hitachi General Hospital

Yurika Yoshikawa, Department of Nursing in Emergency and Critical Care Center(3-2・3-3 ward), Hitachi General Hospital

(○ Principal Investigator)

18. References and Literature List

1)Guidelines for Standard Neurological Treatment: Respiratory Care and Management for

Severe Neurological Diseases. Neurological Therapy Vol. 30, No. 2 (2013)

2)John Robert Bach: Extubation of Patients With Neuromuscular Weakness: American College of Chest Physicians

3)YOKOYAMA Hitoshi, et al.: The Efficacy of Mechanical Insufflator-Exsufflator (MI-E) in ICU Patients with Artificial Ventilation: Japan Society of Intensive Care Medicine Annual Meeting 2016
