# Supplementary material for: Effects of mechanical insufflation-exsufflation on ventilator-free days in intensive care unit subjects with sputum retention; a randomized clinical trial
Source: PLoS One. 2024 May 2;19(5):e0302239. doi: 10.1371/journal.pone.0302239 (PMC11065296; doi:10.1371/journal.pone.0302239)
Supplement: S2 File — (PDF) [file pone.0302239.s003.pdf]

自主臨床研究

集中治療室（ICU）に入室する人工呼吸器装着患者における器械的排痰補助の有用性

研　究　実　施　計　画　書

研究責任者　　日立総合病院　救急集中治療科　中村　謙介
研究担当者　　日立総合病院　救急集中治療科　園生　智弘
日立総合病院　看護局　救命救急センタ（3-2･3-3）係　富岡　真紀子
日立総合病院　看護局　救命救急センタ（3-2･3-3）係　吉川　由利香

作成日
2017年9月13日　計画書案　第1版作成

目　次

0. 概要
1. 研究の背景
2. 研究の目的
3. 対象患者および適格性の基準
4. 研究の方法
5. 観察および検査項目
6. 予想される利益および不利益（副作用）
7. 評価項目（エンドポイント）
8. 有害事象発生時の取扱い
9. 個々の被験者における中止基準
10. 研究実施期間
11. 目標症例数とその設定根拠および統計解析方法
12. 被験者の人権に対する配慮および個人情報の保護の方法
13. 被験者の健康被害への対応と補償
14. 被験者の費用負担
15. 記録の保存と研究結果の公表
16. 研究資金および利益相反
17. 研究実施体制
18. 参考資料・文献リスト

0. 概要

（1）目的

ICUに入室する人工呼吸器装着患者における器械的排痰補助

（Mechanical Insufflator-Exsufflator, MI-E）の有用性について明らかにする。

（2）対象

研究期間内にICU入室となった人工呼吸器装着患者60名

（3）研究の方法

ICUに入室した人工呼吸器管理を必要とした患者の中から頻回の吸引を必要とする患者60名を選出。従来の肺ケアのみを実施した症例（非介入群）30名、従来の肺ケアにMI-Eを使用した症例（介入群）30名に分け、MI-Eの効果を前向きに検証する。調査項目は呼吸器装着日数、再挿管率、気管切開施行率、P/Fratio、CRP、PCT 、ICU在室日数とし、両群間で比較検討する。

（4）評価項目（エンドポイント）

主要評価項目

人工呼吸器装着日数

副次的評価項目

ICU在室日数、死亡率、気管切開施行率、P/Fratio、CRP、PCT

（5）実施予定数：60 人

（6）研究期間：倫理委員会承認後～2019 年 9 月 30 日

1. 研究の背景

器械的排痰補助（Mechanical Insufflator-Exsufflator,MI-E）は、非挿管患者にはマスクを直接あて、また挿管患者には挿管チューブに回路をつなぎ、気道や肺に対し、陽圧と陰圧を付加し、器械的な深呼吸と咳嗽の補助によって気道クリアランスの改善を図ることができる。また無気肺の予防をはじめ、肺や胸郭の可動性やコンプライアンスの維持、深呼吸の補助にも効果的であると言われている。現在MI-Eは、神経筋疾患や脊髄損傷の患者に使用することがガイドラインにおいて推奨されている。[1)2)]

ICUには、意識障害や鎮静状態、疼痛、臥床状態に関連した咳嗽機能低下によって、気道クリアランスの低下する患者が多い。気道クリアランスの低下は、呼吸困難感、呼吸筋疲労、不安定な呼吸状態、人工呼吸管理の長期化を来たす。そしてこれらは、ICU入室期間の延長や筋力低下によるADLの低下、せん妄の助長など患者の機能的、生命予後に大きな影響を及ぼす。

現在、急性期患者へのMI-Eの使用が注目され始め、症例報告や先行研究において有効性[3)]が示唆されている。しかし、気管挿管患者に対するMI-Eの使用報告、有効性に関する先行研究は少ない。当病棟では1年程前よりConfort Cough を使用開始している。非挿管患者と挿管患者に使用をしているが、従来の肺ケアに加えConfort Coughを使用することで、痰のドレナージに有効であると感じることが多い。

そこで今回、人工呼吸器装着患者に焦点をあてMI-Eを使用し、急性期におけるMI-Eの有効性や安全性を明らかにしたいと考えこの研究に取り組みたいと考えた。

2. 研究の目的

ICUに入室する人工呼吸器装着患者における器械的排痰補助

（Mechanical Insufflator-Exsufflator, MI-E）の有用性について明らかにする。

3. 対象患者および適格性の基準

次の選択基準および選択除外基準を満たす、集中治療科が全身管理を行うICUに入室する患者を対象とする。

（1）選択基準

①同意取得時において年齢が18歳以上の患者

②24時間以上人工呼吸器管理をした患者かつ48時間以上の人工呼吸器管理が必要と予測される患者

③喀痰量が多く（概ね1時間に1回以上の吸引が必要）医師が肺理学的療法を必要とする患者

④本研究への参加にあたり十分な説明を受けた後、十分な理解の上、患者家族の自由意思による文書同意が得られた患者

（2）除外基準

研究責任者が被験者として不適当と判断した患者

4. 研究の方法

（1）研究の種類・デザイン

前向き無作為化非盲検化介入試験

(2) 研究のアウトライン

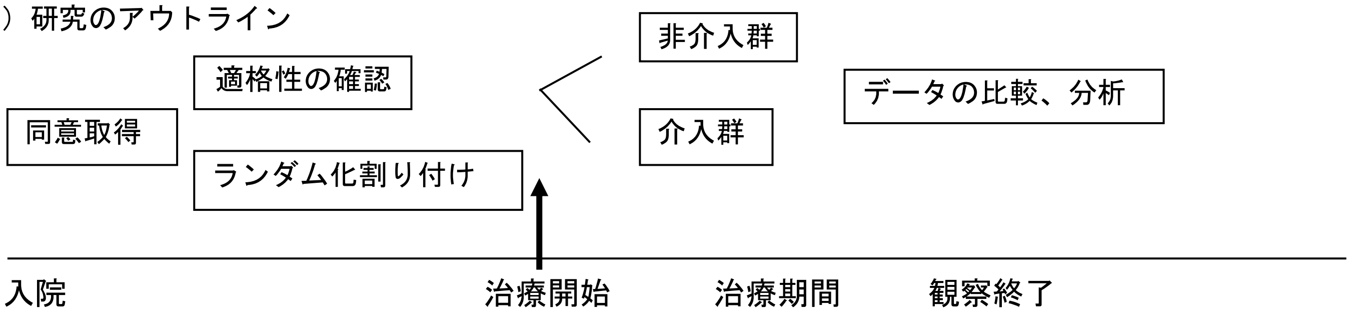

(3) 併用療法についての規定

該当なし

(4) 症例登録、割付の方法

乱数表による無作為割付

(5) 被験者の研究参加予定期間

・参入期間は、倫理委員会承認後から2019年6月30日までの間
・参入基準に該当するすべての患者

5. 観察および検査項目

①患者基本情報：年齢、性別、診断名
②APACHEⅡスコア
③人工呼吸器装着日数
④気管切開施行率
⑤再挿管率
⑥採血データ（CPR、PCT）
⑦血液ガスデータ
⑧ICU滞在日数

6. 予想される利益および不利益（副作用）

(1) 予想される利益

ICU在室日数、人工呼吸器装着日数が減少する可能性がある。
今後の医学の発展に貢献できる可能性がある。

(2) 予想される不利益（副作用）

肺に圧をかけるため、気胸･縦隔気腫などのairleakが生じる可能性があるが、今回使用する40 cmH_2Oの陽圧ではairleakは生じないと考えられる。

7. 評価項目（エンドポイント）

(1) 主要評価項目

人工呼吸器装着日数

（2）副次的評価項目

ICU在室日数、死亡率、気管切開施行率、P/Fratio、CRP、PCT

8. 有害事象発生時の取り扱い

症例報告書に記載する。

9. 個々の被験者における中止基準

（1）患者あるいは代諾者より同意の撤回があった場合
（2）患者あるいは代諾者より治療の変更・中止の申し出があった場合
（3）有害事象の発現（原疾患の増悪、合併症の悪化、新たな疾患の併発等）により、担当医師が試験の継続を不適当と判断した場合

10. 研究実施期間

倫理委員会承認後～2019年9月30日

11. 目標症例数と統計解析方法

（1）目標症例数

60例実施予定

（2）統計解析方法

患者の重症度、検査所見に対して各評価項目を比較する。介入群、非介入群の各２群間比較を検討するため対応のあるｔ検定を使用し分析する。

12. 被験者の人権に対する配慮および個人情報の保護の方法

本研究のすべての担当者は、「ヘルシンキ宣言（2008年10月修正）」および「臨床研究に関する倫理指針（平成20年7月31日改正、以下臨床研究倫理指針）」を遵守して実施する。

研究実施に係る試料等を取扱う際は、被験者の個人情報とは無関係の番号を付して管理し、被験者の秘密保護に十分配慮する。研究の結果を公表する際は、被験者を特定できる情報を含まないようにする。また、研究の目的以外に、研究で得られた被験者の試料等を使用しない。

13. 被験者の健康被害への対応と補償

該当なし。

14. 被験者の費用負担

被験者の費用負担はなし。

15. 記録の保存と研究結果の公表

研究責任者は、研究等の実施に係わる重要な文書を施設内で適切に保存し、研究の中止または終了後10年が経過した日までの間保存し、その後は個人情報に注意して廃棄する。

研究担当者は、本研究の成果を関連学会等において発表することにより公表する。

16. 研究資金および利益相反

該当なし。

17. 研究実施体制

本研究は以下の体制で実施する。

【研究分担者】

〇中村　謙介	株式会社日立製作所	救急集中治療科	センター長
園生　智弘	株式会社日立製作所	救急集中治療科	
富岡　真紀子	株式会社日立製作所	救命救急センタ（3-2･3-3）係	看護師長
吉川　由利香	株式会社日立製作所	救命救急センタ（3-2･3-3）係	看護師

（〇 研究責任者）

18. 参考資料・文献リスト

1)日本神経治療学会治療指針作成委員会：標準的神経治療：重症神経難病の呼吸ケア・呼吸管理とリハビリテーション神経治療　Vol. 30　No. 2（2013）

2)John Robert Bach:Extubation of Patients With Neuromuscular Weakness:American College of Chest Physicians

3)横山仁志他：ICUの人工呼吸器患者における抜管後の器械的排痰補助Mechanical Insufflator-Exsufflator,MI-E）の有用性：日本集中治療医学会学術集会2016
