# Supplementary material for: Effects of mechanical insufflation-exsufflation on ventilator-free days in intensive care unit subjects with sputum retention; a randomized clinical trial
Source: PLoS One. 2024 May 2;19(5):e0302239. doi: 10.1371/journal.pone.0302239 (PMC11065296; doi:10.1371/journal.pone.0302239)
Supplement: S3 File — (DOCX) [file pone.0302239.s004.docx]

(Form No. 2) 　　　　　　　　　　　　　　　　　　　　　　　　　October 16, 2017

Ethics Review Result Report

Head of the Hospital

Mr. Toshiaki Katsuoka

(Ethics Committee)

Committee Chairman Dr. Tsuneo Fujita

Registration Number: 2017-53

Title of Application: The Efficacy of Mechanical Sputum Clearance Assistance in Patients with Mechanical Ventilation in the Intensive Care Unit (ICU)

Principal Investigator: (Hitachi General Hospital) Dr. Kensuke Nakamura

At the Ethics Committee meeting held on October 2, 2017, it was determined as "conditional approval" regarding the ethics review for the above-mentioned study. The result of the re-evaluation is as follows:

1.Decision Result:

Approval

2.Reasons or Recommendations:

No Special Comments

(Form No. 2) October 2, 2017

Ethics Review Result Report

Head of the Hospital

Mr. Toshiaki Katsuoka

(Ethics Committee)

Committee Chairman Dr. Tsuneo Fujita

Registration Number: 2017-53

Title of Application: The Efficacy of Mechanical Sputum Clearance Assistance in Patients with Artificial Ventilation in the Intensive Care Unit (ICU)

Principal Investigator: (Hitachi general Hospital) Dr. Kensuke Nakamura

Regarding the previously submitted ethics review for the study mentioned above, we hereby report the review results as follows:

1.Decision Result:

Conditional Approval

2.Reasons or Recommendations:

[Recommendations]

①It should be stated in the application that there may be disadvantages, such as the inability to receive other forms of treatment due to the allocation.

②The informed consent form should be modified to ensure the consent of both the patient and their legal representative.

※Please submit the revised "Patient Information Sheet" and "Informed Consent Form" to the Ethics Committee Chairman.
