# Supplementary material for: Effects of mechanical insufflation-exsufflation on ventilator-free days in intensive care unit subjects with sputum retention; a randomized clinical trial
Source: PLoS One. 2024 May 2;19(5):e0302239. doi: 10.1371/journal.pone.0302239 (PMC11065296; doi:10.1371/journal.pone.0302239)
Supplement: S4 File — (PDF) [file pone.0302239.s005.pdf]

（様式２号）　　2017年10月16日

倫理審査結果答申書

（病統括）本部長
葛岡　利明　殿

（病統括）倫理委員会
委員長　藤田　恒夫

受付番号　２０１７－５３
申請題名　集中治療室（ICU）に入室する人工呼吸器装着患者における器械的排痰補助の有用性
実施責任者　（日病）中村　謙介

2017年10月2日に開催の（病統括）倫理委員会において、「条件付承認」となっておりました上記に係る倫理審査について、再審査の結果を下記の通り答申いたします。

記

１．判定結果<br><br>承　認
２．理由又は勧告事項<br><br>【勧告事項】<br><br>特に無し。

（様式２号）　　　　　　　　　　　　　　　　　　　　　　2017 年 10 月 2 日

倫理審査結果答申書

（病統括）本部長
葛岡　利明　殿

（病統括）倫理委員会
委員長　藤田　恒夫

受付番号　２０１７－５３
申請題名　集中治療室（ICU）に入室する人工呼吸器装着患者における器械的排痰補助の有用性
実施責任者　（日病）中村　謙介

先に申請のあった上記に係る倫理審査について審査結果を下記の通り答申いたします。

記

1．判定結果<br><br>条件付き承認
2．理由又は勧告事項<br><br>【勧告事項】<br><br>① 割付が行なわれることによって他方の治療が受けられないデメリットなど記載のこと<br>② 同意書は本人と代諾者の同意が取れる様式にすること。<br><br>※上記を修正した「患者説明書」「同意書」を倫理委員長に提出してください。
