# Supplementary material for: Effects of mechanical insufflation-exsufflation on ventilator-free days in intensive care unit subjects with sputum retention; a randomized clinical trial
Source: PLoS One. 2024 May 2;19(5):e0302239. doi: 10.1371/journal.pone.0302239 (PMC11065296; doi:10.1371/journal.pone.0302239)
Supplement: Attachment — Submitted filename: comment .docx [file pone.0302239.s007.docx]

This study is one of the few RCT studies in the intensive care field of M I-E and the results are scientific importance.

Several matters are commented on below that require mentioning.

1. The exclusion criteria do not contain pneumothorax, which is the main adverse event when using M I-E.
2. More than 80% of the subjects are male, but how many have a smoking history or chest C T that may have a bullae?　The MI-E pressure of 40 cm H2O was performed in all the same cases, but it is necessary to mention how it was managed and whether there were no adverse events.
3. In line 106 of the Randomisation and procedures, the word "respiratory chest physiotherapy" are not often used and should be corrected to either "chest physiotherapy" or "respiratory physiotherapy".
4. Lines 227-229; the reference to 40 mmHg is cited as justification for setting MI-E pressure in this study, but the unit of 40 mmHg is 54 cm H2O when calculated in cm H2O, which may mislead the reader. Reference: <https://pubmed.ncbi.nlm.nih.gov/25492956/>
5. The structure of the discussion lacks logic.

It would be better to describe what was expected in this hypothesis, what were the results, what were the factors, sample size and other factors due to the study design have been mentioned, but are there other factors, are there any respiratory physiological factors, and should include reference to medical factors.
