# Supplementary material for: Effects of mechanical insufflation-exsufflation on ventilator-free days in intensive care unit subjects with sputum retention; a randomized clinical trial
Source: PLoS One. 2024 May 2;19(5):e0302239. doi: 10.1371/journal.pone.0302239 (PMC11065296; doi:10.1371/journal.pone.0302239)
Supplement: Attachment — Submitted filename: Review WS - PONE-D-23-25920.docx [file pone.0302239.s008.docx]

**Review WS PONE-D-23-25920**

Dear authors,

Interesting study that is adding to current information on MI-E in invasively ventilated patients.

There are some aspects that could improve the current manuscript.

1. The emailadress of the corresponding author does not provide any information on who this is or what institution is behind this. Could this be checked and changed into a professional emailadress?
2. The registration number in UMIN, nor the title of the manuscript provide a preregistration on the site. It is therefore not possible to see if the analysis is performed according to the predefined plan. Possible a number is changed? Could you please have a closer look to make this correct?
3. Two systems have been used to refer to references. For example reference number 4 in line 52 and 59.
4. Check spelling author in line 68.
5. Some wordings or sentences are incorrect. I would suggest to have a native speaker check the manuscript.
6. Currently there is no clarification regarding a power calculation to show significance. Please clarify how the study was set up. Was the number of 30 per group a practical decision or calculated?
7. The hypothesis is not included in the manuscript. Please add after the aim.
8. The inclusion criteria are not clear with regard to the suctioning. The indication of requiring suctioning is clear, but not for how long or how many hours this should have been the case before inclusion. In addition could the authors clarify how the professionals were trained with regard to endotreacheal suctioning and the indication to initiate endotracheal suctioning? Currently there is a possibility of selection bias.
9. The informed consent procedure should be clarified. Were patients asked informed consent while being intubated or their nearests? Sentence no 90 does give the impression. Was informed consent of the patient asked after extubation? Please clarify the informed consent procedure.
10. The randomization procedure should be clarified for the aspect of allocation concealment and how this was ensured by using the FileMaker pro 16- system. The current procedure could introduce selection bias.
11. Could the authors clarify the intervention with regard to indication for use and frequency of use per day? Currently it could be that one patient received the MI-E every hour and another only a few times a day.
12. Additionally it is unclear how patients were humidified during mechanical ventilation and what other airway care interventions were performed, for example nebulization therapy or manual hyperinflation. In the current description there is a risk of performance bias.
13. Could the authors describe what they defined as adverse events in this study?
14. Reference include many articles with regard to patient with neuromuscular disease. In the last year more studies have been published with regard to MI-E in invasively ventilated patients. Please have another reading of the literature and integrate these in the discussion. For example the Scoping review with an overview of adverse events in invasively ventilated patients (PMID: 35610033) and the study of Marti (PMID: 36041752) and Wu (PMID: 36041752).
15. The Kaplan Meyer is nice to add, but could be made more clear by a distinction between the groups. Maybe a continuous line and dots or stripes would be more clear for reading?
