# Supplementary material for: Effects of mechanical insufflation-exsufflation on ventilator-free days in intensive care unit subjects with sputum retention; a randomized clinical trial
Source: PLoS One. 2024 May 2;19(5):e0302239. doi: 10.1371/journal.pone.0302239 (PMC11065296; doi:10.1371/journal.pone.0302239)
Supplement: Attachment — Submitted filename: Response to Reviewers.docx [file pone.0302239.s009.docx]

Dear Dr. Lascarrou,

Thank you for your e-mail regarding our manuscript entitled “Effects of mechanical insufflation-exsufflation on ventilator-free days in intensive care unit subjects with sputum retention; A randomized clinical trial”, and the valuable comments of the three Reviewers. Please find attached our revised manuscript as well as a point-by-point responses to the Reviewers’ comments. We apologize for the initial submission, which was not written in standard English. The manuscript was proofread by a native English speaker to enhance its readability. I have received the following comment from Reviewer 3: "The email address of the corresponding author does not provide any information on who this is or what institution is behind this. Could this be checked and changed into a professional email address?" We apologize for the non-formal e-mail address, which was changed to a professional e-mail address in the revised manuscript to clarify the affiliation. Furthermore, since the corresponding author is affiliated with two institutions, his affiliation was updated to reflect this. We changed the following e-mail address from “[mamashockpapashock@yahoo.co.jp](mailto:mamashockpapashock@yahoo.co.jp)” to “[nakamura.ken.kl@yokohama-cu.ac.jp](mailto:nakamura.ken.kl@yokohama-cu.ac.jp)” We also added the following as affiliations:“Department of Critical Care Medicine, Yokohama City University Hospital, Kanagawa, Japan” PACE was used to ensure that the figures complied with PLOS ONE’s submission guidelines.

We consider the revised manuscript to be a suitable response to the Reviewers’ comments and it has been significantly improved over the initial submission. We trust that it is now suitable for publication in PLOS ONE. Thank you in advance for your kind consideration of this manuscript.

Sincerely yours,

Shota Kubota

Department of Emergency and Critical Care Medicine, Hitachi General Hospital, Ibaraki, Japan

**RESPONSE TO REVIEWER 1:**

We wish to express our strong appreciation to the Reviewer for their insightful comments, which have helped us to significantly improve the quality of the revised manuscript.

**Comment 1:** The introduction should better reflect the current knowledge on the topic. Reporting results from observational studies in intubated patients in which mechanical insufflation-exsufflation was tested to hasten weaning from mechanical ventilation (ref 14 and 15) may be more appropriate than reporting the results from Gonçalves and colleagues who tested mechanical insufflation-exsufflation after extubation (not “peri-extubation” as stated line 68) to avoid reintubation.

**Response:** We appreciate the Reviewer’s comment. Studies that used mechanical insufflation-exsufflation to hasten weaning from mechanical ventilation in intubated patients were cited in the revised manuscript instead of those that used mechanical insufflation-exsufflation after extubation.

In accordance with the Reviewer’s comment, we changed the following text from

“Previous studies demonstrated the efficacy of MI-E in extubation failure in critical care settings. Gonalves MR et al. reported that the peri-extubation use of MI-E increased the probability of successful extubation.”

to

“Observational trials compared the usefulness of MI-E for ventilated subjects: the use of MI-E on ventilated subjects with neuromuscular diseases facilitated weaning off the ventilator and prevented reintubation.” (lines 69-71)

Moreover, in accordance with the Reviewer’s comment, when referring to the findings reported by Gonçalves and colleagues, the expression “peri-extubation” was changed to “after extubation”. We apologize for the ambiguous wording.

**Comment 2:** What is the airway suctioning protocol in the unit? I it “on-demand” or “scheduled?

**Response:** We wish to thank the Reviewer for this comment. We agree that more precise details were needed on the frequency and method of suction. Suctioning was performed on-demand. Although subjects who required suctioning at least once every hour at the start of the study were included in the research, there was no upper limit to the number of times. Moreover, if it was not possible to suction airway secretions after inclusion, and no further suctioning of airway secretions was expected to be possible based on a physical examination and auscultation, suctioning was allowed to occur less frequently than once an hour. This may have affected the results obtained. However, performing additional suctioning on patients who do not produce further airway secretions despite suctioning may result in more side effects than benefits; therefore, we were unable to maintain the same frequency for all patients. We documented this as a limitation in the Discussion section.

In accordance with the Reviewer’s comment, we changed the following sentences from (lines 116-118)

“However, if they no longer needed to suction frequently based on the clinical assessment after study inclusion, they were allowed to suction less often than once every hour.”

to

“Suctioning was performed on-demand by the nurse in charge who fully understood the protocol of the study and had received adequate training and practical experience with suctioning. Although subjects who required suctioning at least once every hour at the start of the study were included in the research, there was no upper limit to the number of times. Moreover, if it was not possible to suction airway secretions after inclusion and no further suctioning of airway secretions was expected to be possible based on a physical examination and auscultation, suctioning was allowed to occur less frequently than once an hour.” (line 131-139)

and

“In the MI-E group, if there were no more airway secretions to be cleared even after using MI-E, suctioning and the use of MI-E were permitted at intervals exceeding one hour.” (line 146-148)

Furthermore, the following sentences were added to the Discussion section.

“It was difficult to establish a fixed number of suctions per hour because additional suctioning or the use of MI-E in the absence of further cleared airway secretions may have led to potential side effects without corresponding benefits. However, variability in the frequency of suctioning and MI-E sessions that individual patients received each day may have affected the outcomes.” (line 334-339)

**Comment 3:** For how many hours should airway be suctioned more than once per hour before inclusion? What was the time limit for inclusion? Could a patient be included after 10 days of mechanical ventilation, which may have biased the results?

**Response:** We wish to thank the Reviewer for this comment. We apologize for the unclear description of patient inclusion criteria. To clarify which patients were included and at what specific times, the following sentences were added to the Methods section.

“Patients were included if they required suctioning more than once every hour for the first 24 hours after intubation. If they did not require suctioning once every hour in the initial 24 hours, but needed suctioning at least once every hour in the subsequent 24 consecutive hours, they were included at that point. There was no specific time limit on inclusion regarding the number of days elapsed after intubation.” (line 91-96)

As suggested, there may be a potential for bias due to variations in the period between intubation and inclusion. Therefore, the following sentences were added to the Discussion section.

“Suction frequency was initially low immediately after intubation; however, patients were included if they required suctioning at a rate of once per hour or more within the subsequent continuous 24-hour period. This variation in the time between intubation and inclusion may have potentially affected the results obtained herein.” (line 339-342)

**Comment 4:** Was there a standardized weaning protocol? In the absence of a weaning protocol, mechanical insufflation-exsufflation may have delayed extubation. Indeed, mechanical insufflation-exsufflation increases the amount of secretions which may have led the attending physician to delay extubation due to excessive secretions.

**Response:** We appreciate the Reviewer’s comment on this point. In the present study, there was no specific weaning protocol defined in the research protocol. Weaning decisions were made at the discretion of the attending physician and ICU staff. However, it is important to note that the majority of weaning decisions followed the Ventilator Weaning Program published by the Japanese Society of Intensive Care Medicine. Part of the description and a citation of these guidelines are provided below.

“Extubation is attempted in patients with successful SBT; the five criteria for the initiation of SBT are as follows: 1)Adequate oxygenation, defined as SpO_2_ >90% when FIO_2_ is <0.5 and PEEP <8 cmH_2_O. 2)Hemodynamic stability, with no acute myocardial ischemia or severe arrhythmia, a heart rate ≤140 bpm, and only small doses of vasoactive agents are employed; dopamine ≤5 μg/kg/min, dobutamine ≤5 μg/kg/min, noradrenaline ≤0.05 μg/kg/min. 3) Adequate ventilation effort, with ventilation volume >5 ml/kg per breath, minute ventilation <15 L/min, and absence of respiratory acidosis (pH >7.25). 4) No abnormal breathing patterns, excessive use of respiratory support muscles, or paradoxical breathing. 5) General condition is stable with no fever, severe electrolyte imbalances, severe anemia, or severe fluid overload. The success criteria for SBT were defined as the following conditions being met: 1) Respiratory rate <30 breaths/min. 2) No significant decline compared to before SBT starting, such as SpO_2_ ≥94% or PaO_2_ ≥70 mmHg. 3) Heart rate <140 bpm with no evidence of new arrhythmia or myocardial ischemia. 4) No excessive increase in blood pressure.”

“The Japanese Society of Intensive Care Medicine, the ventilator weaning protocol, <https://www.jsicm.org/pdf/kokyuki_ridatsu1503a.pdf>”

Although there was no pre-defined weaning protocol, the following description on the weaning method employed in the present study was added to the Methods section.

“Daily assessments of the ability to breathe with pressure support ventilation were performed when the positive end-expiratory pressure and fraction of inspiratory oxygen levels were lower than the day before. Additionally, the ventilator was switched to pressure support ventilation if the attending physician considered the patient sufficiently awake to breathe with pressure support ventilation. Extubation was attempted in patients with a successful spontaneous breathing trial (SBT). The ICU attending physician made the final decision regarding whether to extubate the patient.” (line 122-129)

Furthermore, in accordance with the Reviewer’s comment, the following sentences on this point were added to the Discussion section.

“Since 3blinding was not possible due to the nature of the intervention, there may have been performance, reporting, and selection biases. Suctioning may be performed more effectively and carefully in the MI-E group. MI-E increased the amount of secretions, which may have led the attending physician to delay extubation due to excessive secretions.” (line 330-335)

**Comment 5:** What is the tracheostomy policy? Is it early systematic tracheostomy, or late tracheostomy in selected patients?

**Response:** We thank the Reviewer for this comment. We did not actively perform early tracheostomy. Tracheostomy was performed when endotracheal intubation was anticipated to exceed 14 days. Therefore, the following sentence was added to the Methods section.

“Tracheostomy was performed if the ventilator duration was expected to be more than 14 days.” (line 129-130)

The following information was added to the Results section on the actual duration from intubation to tracheostomy.

“The median duration from intubation to tracheostomy was 12 (IQR, 9–19) in the MI-E group and 12 (IQR, 6–15) in the control group; P=0.85.” (line 228-229)

**Comment 6:** Who performed the mechanical insufflation-exsufflation? Please detail the protocol (the sentence “mechanical insufflation-exsufflation was used 10 time for each sputum aspiration”, lines 112-113 is not clear), including its weaning (line 116 is not clear).

**Response:** We wish to thank the Reviewer for this comment. Mechanical insufflation-exsufflation was performed by the nurse in charge who fully understood the protocol of the study and had received adequate training and practical experience with MI-E. In accordance with the Reviewer’s comment, we changed sentences on the MI-E protocol and its weaning from

“In both groups, subjects who required suctioning at least once every hour at the start of the study were included in the research. However, if they no longer needed to suction frequently based on the clinical assessment after study inclusion, they were allowed to suction less often than once every hour.”

to

“Suctioning was performed on-demand by the nurse in charge who fully understood the protocol of the study and had received adequate training and practical experience with suctioning. Although subjects who required suctioning at least once every hour at the start of the study were included in the research, there was no upper limit to the number of times. Moreover, if it was not possible to suction airway secretions after inclusion and no further suctioning of airway secretions was expected to be possible based on a physical examination and auscultation, suctioning was allowed to occur less frequently than once an hour.” (line 131-139)

and

“In the MI-E group, if there were no more airway secretions to be cleared even after using MI-E, suctioning and the use of MI-E were permitted at intervals exceeding one hour.” (line 146-148)

**Comment 7:** The secondary outcomes should be listed explicitly.

**Response:** We wish to thank the Reviewer for this comment. We apologize for the unclear method of presentation, which was edited in the revised manuscript by a native English speaker as follows.

“Secondary outcomes were the duration of mechanical ventilation up to day 28, the ICU length of stay, the mortality rate, and the tracheostomy rate. Secondary outcomes, except for mortality, were analyzed exclusively for patients who survived.” (line 166-169)

**Comment 8:** Respectfully, “the ability to remain free from mechanical ventilation for 24 hours” is not the same between extubation and tracheostomy (lines 130-132). Airway suctioning is easier in tracheostomized patients than in extubated patients.

**Response:** We wish to express our deep appreciation to the Reviewer for their insightful comment on this point, with which we agree. In the present study, it was necessary to aggregate the duration after extubation and after tracheostomy without mechanical ventilation through some method. A method that differentiates and aggregates the duration after extubation and after tracheostomy without mechanical ventilation separately would be ideal. However, to combine them, there was no alternative but to treat them equivalently. Since the points raised by the Reviewer are valid, the following sentences were added as a limitation in the Discussion section.

“In the present study, ventilator-free days for patients following extubation and tracheotomy without mechanical ventilation were counted as equivalent. While tracheotomized patients are more likely to clear airway secretions than extubated patients, there is no feasible method to unify or convert ventilator-free days between extubated and tracheotomized patients. Therefore, we were compelled to treat ventilator-free days in extubated and tracheotomized patients as equivalent.” (line 346-352)

**Comment 9:** Please detail the adverse events reviewed.

**Response:** We thank the Reviewer for this comment. We agree that more detailed descriptions of both potential adverse events and the actual adverse events that occurred are needed. In accordance with the Reviewer’s comment, we changed the sentence from

“There were no adverse events associated with this study.”

to

“While some patients had hemodynamic instability or arrhythmia at the time of inclusion, no new cases of hemodynamic instability or arrhythmia occurred with the use of MI-E. The use of MI-E did not lead to new cases of lung injury due to barotrauma, volutrauma, atelectrauma, or biotrauma. No other apparent adverse effects attributed to MI-E occurred in the present study, such as pneumothorax, hemoptysis, oxygen desaturation, and mucus plugging.” (line 232-237)

The following descriptions on potential adverse events were also added to the Discussion section.

“In 13 studies that used MI-E, 10 found no adverse events, while 3 reported oxygen desaturation, hemodynamic variation, pneumothorax, mucus plugging, hemoptysis, and chest pain as adverse events.[17] In the present study, 33% of patients in the control group and 48% in the MI-E group had a history of smoking. All patients had undergone chest CT, and the percentage of patients with bullae in the lungs was 62% in the control group and 59% in the MI-E group. Pneumothorax did not occur in patients, even in those with a history of smoking or bullae.” (line 302-309)

**Comment 10:** For statistical analysis, please specify the expected number of ventilator free days, in the control group to better understand whether the sample size calculation was realistic.

**Response:** We appreciate the Reviewer’s comment on this point. Regarding the expected number of ventilator-free days in the control group, we based our calculations on the previous duration in our institution. In the medical records of Hitachi General Hospital, the duration of mechanical ventilation for respiratory disease patients who received mechanical ventilation for 48 hours or longer between 2015 to 2017 was 7 days (IQR, 4–15). Ideally, we would have preferred data on patients requiring suction at least once every hour, similar to the criteria of the present study. However, since there were no studies conducted on the same set of patients, we focused on respiratory disease patients as the most similar target group. Furthermore, by narrowing the focus to patients requiring the suctioning of sputum at least once every hour, the duration of mechanical ventilation was considered to be even longer than that in all respiratory disease patients. Therefore, in the control group in the present study, we considered a median duration of mechanical ventilation of 7 days or longer and estimated the expected number of ventilator-free days up to 28 day in the control group to be 21 days or less. The present results showed that the number of ventilator-free days was 21 in the MI-E group and 18 in the control group. These results were in line with our initial expectations.

**Comment 11:** For primary outcome, it is not clear which statistical test was used (Mann-Whitney? Cox model?). Additionally, the statistical analysis paragraph can easily be shortened and simplified to be more readable.

**Response:** We wish to thank the Reviewer for this comment. We apologize for the confusion. The Mann-Whitney test was used for the primary outcome. The following changes were made in the revised manuscript, which was reviewed by a native speaker of English.

“To analyze the primary outcome and secondary outcomes, except for mortality and tracheostomy rates, the Mann-Whitney U test was used with 95% CIs for superiority. To analyze mortality and tracheostomy rates as well as subject characteristics, the chi-square test or Fisher’s exact test were used where appropriate. The duration of mechanical ventilation up to day 28 in surviving subjects was compared using Kaplan-Meier survival curves. Survival times were calculated from the time of randomization until the time of death or loss to the follow-up. All statistical tests were two-sided, with a significance level of p <0.05. All analyses were performed using R software, version 2.7-1.” (line 190-198)

**Comment 12:** The results are overinterpreted. The ventilator-free days are not “slightly higher” in the mechanical insufflation-exsufflation, they were not different. “Secondary outcomes […] were better in the mechanical insufflation-exsufflation group” is not true, they were not different. Additionally, the tracheostomy rate among survivors is not clear. According to Table 2, in the intervention group, 6 out of 27 patients died, so 21 patients survived. Therefore, the tracheostomy rate should be 3/21, not 3/22.

**Response:** We appreciate the Reviewer’s comment on this point. We apologize for the overinterpretation of the results. All overinterpreted statements in the Abstract, Results, Discussion, and Conclusion sections were revised to accurately reflect the lack of significance. The tracheostomy rate was 3/21 and this error has been corrected in accordance with the Reviewer’s comment. Information in Table 2 has been updated. (line 239)

**Comment 13:** In the discussion section, please also discuss the fact that the absence of difference could be due to the population selected which could not be the good target, or to the absence of efficacy of the device.

**Response:** We wish to thank the Reviewer for this comment. We considered several reasons for the lack of significant differences in the present study. Even if there were improvements in airway resistance or lung compliance, we considered the possibility that the reasons for requiring mechanical ventilation, such as pneumonia, cardiac arrest, or sepsis, were not fundamentally improved. We also entertained the possibility that the effectiveness of MI-E may have been insufficient or that there may have been potential adverse events associated with its use. The following sentences were added to the Discussion section.

“While the use of MI-E may reduce airway resistance or enhance compliance [7,8], it does not directly address the underlying medical factors (such as pneumonia, cardiac arrest, sepsis, and surgery) that necessitated intubation. This may explain why the number of ventilator-free days was not significantly different. A pressure of ±40 cmH_2_O may have been insufficient for patients with excessive sputum production, such as those examined in the present study. On the other hand, although MI-E was effective, potential adverse events, such as barotrauma or atelectrauma, may have affected the results obtained.” (line 256-263)

**Comment 14:** Does the “the number of days of ventilatory management” means “duration of mechanical ventilation”?

**Response:** We thank the Reviewer for this comment. The expression “the number of days of ventilatory management” indicated “the duration of mechanical ventilation”. We apologize for any confusion. All relevant phrases were standardized to “the duration of mechanical ventilation”.

**Comment 15:** English editing would greatly improve the reading of the manuscript.

**Response:** We appreciate the Reviewer’s comment on this point. The revised text was reviewed by a native speaker of English.

We wish to thank the Reviewer again for their valuable comments.

**RESPONSES TO REVIEWER 2:**

We wish to express our appreciation to the Reviewer for their insightful comments, which have helped us to significantly improve the quality of the revised manuscript.

**Comment 1:** The exclusion criteria do not contain pneumothorax, which is the main adverse event when using MI-E.

**Response:** We appreciate the Reviewer’s comment on this point. Patients who were considered to have underlying conditions that may be exacerbated by MI-E were excluded by the attending physician. The following sentence in the Methods section was changed from

“Patients who met the following criteria were excluded from the study: pregnant women, […], and cases that were deemed inappropriate by the attending physician.”

to

“Patients who met the following criteria were excluded from the study: pregnant women, […], and patients considered by the attending physician to have a coexisting condition that is exacerbated by MI-E, such as pneumothorax, empyema with fistula, or hemoptysis.” (line 96-101)

**Comment 2:** More than 80% of the subjects are male, but how many have a smoking history or chest C T that may have a bullae? The MI-E pressure of 40 cm H2O was performed in all the same cases, but it is necessary to mention how it was managed and whether there were no adverse events.

**Response:** We appreciate the Reviewer’s comment on this point. In the present study, the collection of smoking history and chest CT scans were not specified in the study protocol. However, at Hitachi General Hospital, routine smoking history inquiries are conducted upon admission. Therefore, we retrospectively examined the smoking history of all patients. Additionally, since all patients included in the present study had undergone chest CT imaging, we retrospectively assessed the percentage of patients with lung bullae. The results obtained showed that 33% of patients in the control group and 48% in the MI-E group had a history of smoking, while 62% in the control group and 59% in the MI-E group had bullae in the lungs.

We also provided more detailed information on how to connect the MI-E machine and adjust pressure settings in the Methods section.

“When the MI-E COMFORT COUGH-Ⅱ is directly connected to the endotracheal tube and the pressure is set to 40 cmH_2_O, it applies both positive and negative pressures of 40 cmH_2_O during inhalation and exhalation, respectively, inside the endotracheal tube.” (line 141-144)

More detailed descriptions of actual adverse events were also added to the Results section.

“While some patients had hemodynamic instability or arrhythmia at the time of inclusion, no new cases of hemodynamic instability or arrhythmia occurred with the use of MI-E. The use of MI-E did not lead to new cases of lung injury due to barotrauma, volutrauma, atelectrauma, or biotrauma. No other apparent adverse effects attributed to MI-E occurred in the present study, such as pneumothorax, hemoptysis, oxygen desaturation, and mucus plugging.” (line 232-237)

Based on previous studies, the following sentences on the adverse event of pneumothorax, in connection with a smoking history or bullae were added to the Discussion section.

“In 13 studies that used MI-E, 10 found no adverse events, while 3 reported oxygen desaturation, hemodynamic variation, pneumothorax, mucus plugging, hemoptysis, and chest pain as adverse events.[17] In the present study, 33% of patients in the control group and 48% in the MI-E group had a history of smoking. All patients had undergone chest CT, and the percentage of patients with bullae in the lungs was 62% in the control group and 59% in the MI-E group. Pneumothorax did not occur in patients, even in those with a history of smoking or bullae.” (line 302-309)

**Comment 3:** In line 106 of the Randomisation and procedures, the word "respiratory chest physiotherapy" are not often used and should be corrected to either "chest physiotherapy" or "respiratory physiotherapy".

**Response:** We thank the Reviewer for this comment. This expression was standardized to “respiratory physiotherapy”.

**Comment 4:** Lines 227-229; the reference to 40 mmHg is cited as justification for setting MI-E pressure in this study, but the unit of 40 mmHg is 54 cm H2O when calculated in cm H2O, which may mislead the reader. Reference: <https://pubmed.ncbi.nlm.nih.gov/25492956/>

**Response:** We strongly agree with the Reviewer’s comment. We apologize for any confusion. Units need to be consistently aligned and were standardized to cmH_2_O. The following changes were made in the description from

“Pressures of 40 mmHg to –40 mmHg are usually most effective and best tolerated.”

to

“Since another study demonstrated that pressures of ±54 cmH_2_O were tolerated well, we assumed that a pressure of ±40 cmH_2_O may also be tolerated.” (line 298-299)

**Comment 5:** The structure of the discussion lacks logic. It would be better to describe what was expected in this hypothesis, what were the results, what were the factors, sample size and other factors due to the study design have been mentioned, but are there other factors, are there any respiratory physiological factors, and should include reference to medical factors.

**Response:** We wish to express our deep appreciation to the Reviewer for their insightful comment on this point. The expected hypothesis and the results obtained were added to the beginning of the Discussion section as follows.

“The hypothesis of the present study was that the additional use of MI-E in conventional pulmonary care shortens the duration of mechanical ventilation in subjects with high airway secretions. However, the use of MI-E before each direct tracheal suction did not significantly reduce the number of ventilator-free days by day 28. In addition, no significant differences were observed in the duration of mechanical ventilation, the ICU length of stay, the mortality rate, or the tracheostomy rate between the MI-E and control groups.” (line 247-253)

In the present study, we considered several factors that may have contributed to the lack of significant differences. Even if there were improvements in airway resistance or lung compliance, we considered the possibility that fundamental reasons for requiring mechanical ventilation, such as pneumonia, cardiac arrest, or sepsis, were not improved. We also entertained the possibility that the effectiveness of MI-E may have been insufficient or that there may have been potential adverse events associated with its use. The following sentences were added to the Discussion section.

“While the use of MI-E may reduce airway resistance or enhance compliance [7,8], it does not directly address the underlying medical factors (such as pneumonia, cardiac arrest, sepsis, and surgery) that necessitated intubation. This may explain why the number of ventilator-free days was not significantly different. A pressure of ±40 cmH_2_O may have been insufficient for patients with excessive sputum production, such as those examined in the present study. On the other hand, although MI-E was effective, potential adverse events, such as barotrauma or atelectrauma, may have affected the results obtained.” (line 256-263)

Thank you again for your comments on our manuscript. We trust that the revised manuscript will now be considered suitable for publication.

**RESPONSES TO Dr. Willemke Stilma; REVIEWER 3:**

We wish to express our appreciation to the Reviewer for their insightful comments, which have helped us to significantly improve the quality of the revised manuscript.

**Comment 1:** The emailadress of the corresponding author does not provide any information on who this is or what institution is behind this. Could this be checked and changed into a professional emailadress?

**Response:** We wish to thank the Reviewer for this comment. We apologize for the non-formal e-mail address, which was changed to a professional e-mail address in the revised manuscript to clarify the affiliation. Furthermore, since the corresponding author is affiliated with two institutions, his affiliation was updated to reflect this. We changed the following e-mail address from

[mamashockpapashock@yahoo.co.jp](mailto:mamashockpapashock@yahoo.co.jp)

to

[nakamura.ken.kl@yokohama-cu.ac.jp](mailto:nakamura.ken.kl@yokohama-cu.ac.jp) (line 16)

We also added the following as affiliations:

“Department of Critical Care Medicine, Yokohama City University Hospital, Kanagawa, Japan” (line 12-13)

**Comment 2:** The registration number in UMIN, nor the title of the manuscript provide a preregistration on the site. It is therefore not possible to see if the analysis is performed according to the predefined plan. Possible a number is changed? Could you please have a closer look to make this correct?

**Response:** We thank the Reviewer for this comment. We apologize for any inconvenience. The trial registered in UMIN is named “Effect of mechanical sputum assistance for ventilated patients in intensive care unit”. However, the UMIN number mentioned in the manuscript is correct. When searching the site for UMIN Clinical Trials, we found our registration by searching for the UMIN number previously included in the manuscript. The internet site used for the search and the UMIN number are shown below.

<https://center6.umin.ac.jp/cgi-open-bin/ctr_e/index.cgi?function=02>

registration number UMIN000033980

We sincerely apologize again for the discrepancy in the registered name.

**Comment 3:** Two systems have been used to refer to references. For example reference number 4 in line 52 and 59.

**Response:** We thank the Reviewer for this comment. We apologize for the inconsistent representation. The citation system for all references was standardized in the revised manuscript.

**Comment 4:** Check spelling author in line 68.

**Response:** We appreciate the Reviewer’s comment on this point. It was not Gonalves, but Gonçalves. We apologize for this error, which was corrected in the revised manuscript.

**Comment 5:** Some wordings or sentences are incorrect. I would suggest to have a native speaker check the manuscript.

**Response:** We appreciate the Reviewer’s comment on this point. We apologize for our errors. The revised manuscript was reviewed by a native speaker of English.

**Comment 6:** Currently there is no clarification regarding a power calculation to show significance. Please clarify how the study was set up. Was the number of 30 per group a practical decision or calculated?

**Response:** We wish to thank the Reviewer for this comment. The sample size was calculated. In a previous study performed in Hitachi General Hospital, the standard deviation of ventilator-free days up to 28 days for subjects ventilated for more than 3 days in the ICU was 7.6 days. Since the standard deviation was expected to decrease when subjects were limited to those with high sputum retention, we assumed the baseline standard deviation of ventilator-free days in the present study to be 5 days. To provide 80% power to detect a reduction of 4 ventilator-free days in the MI-E group for the primary endpoint, with a two-sided 5% type 1 error rate, a sample size of 25 individuals was required for each group. To account for withdrawals during the present study, we set the sample size of participants as 30 per group.

We planned to terminate enrollment once we reached the target sample size or in June 2019. Enrollment concluded in June 2019 before reaching the targeted sample size. When we planned the research, the sample size was calculated; however, due to the smaller number of eligible patients than anticipated, we were unable to reach the intended sample size as a result.

**Comment 7:** The hypothesis is not included in the manuscript. Please add after the aim.

**Response:** We thank the Reviewer for this comment. In accordance with this comment, the following sentence was added after the aim in the Introduction section.

“The hypothesis of the present study was that the additional use of MI-E in conventional pulmonary care shortens the duration of mechanical ventilation in subjects with high airway secretions.” (line 75-78)

**Comment 8:** The inclusion criteria are not clear with regard to the suctioning. The indication of requiring suctioning is clear, but not for how long or how many hours this should have been the case before inclusion. In addition could the authors clarify how the professionals were trained with regard to endotreacheal suctioning and the indication to initiate endotracheal suctioning? Currently there is a possibility of selection bias.

**Response:** We wish to express our deep appreciation to the Reviewer for the insightful comment on this point. We apologize for the unclear description of patient inclusion criteria. To clarify which patients were included and at what specific times, the following sentences were added to the Methods section.

“Patients were included if they required suctioning more than once every hour for the first 24 hours after intubation. If they did not require suctioning once every hour in the initial 24 hours, but needed suctioning at least once every hour in the subsequent 24 consecutive hours, they were included at that point. There was no specific time limit on inclusion regarding the number of days elapsed after intubation.” (line 91-96)

There may have been a potential for bias due to variations in the period between intubation and inclusion. Therefore, the following sentences were added to the Discussion section.

“Suction frequency was initially low immediately after intubation; however, patients were included if they required suctioning at a rate of once per hour or more within the subsequent continuous 24-hour period. This variation in the time between intubation and inclusion may have potentially affected the results obtained herein.” (line 339-342)

Prior to participating in the present study, nurses received consistent training on the use of MI-E, but had not received uniform training in suctioning. As suggested, there is a possibility of selection bias due to the lack of uniform training in suctioning. Additionally, the indication to initiate endotracheal suctioning was not clearly defined and was selected by the nurse based on a comprehensive assessment, including physical observations, auscultation findings, and whether airway secretions were expectorated during the previous suctioning procedure. This may also have acted as a selection bias. Therefore, the following sentences were added to the Discussion section.

“Furthermore, not all nurses had received uniform training in suctioning; therefore, there was a potential for selection bias. The timings of suctioning and nebulization were selected by nurses based on a comprehensive assessment, including physical and auscultation findings and whether airway secretions had been cleared during the previous suctioning. However, due to the absence of standardized rules for the timing of suctioning and nebulization, there was a potential for selection bias.” (line 313-318)

**Comment 9:** The informed consent procedure should be clarified. Were patients asked informed consent while being intubated or their nearests? Sentence no 90 does give the impression. Was informed consent of the patient asked after extubation? Please clarify the informed consent procedure.

**Response:** We appreciate the Reviewer's comment on this point. We apologize for the inadequate description. In accordance with this comment, we added the following sentences to the Methods section.

“Since the present study involved intubated patients, written informed consent was obtained from their legal representatives at the time of inclusion for the majority of patients. Whenever possible, informed consent was also obtained from patients after extubation.” (line 108-111)

**Comment 10:** The randomization procedure should be clarified for the aspect of allocation concealment and how this was ensured by using the FileMaker pro 16- system. The current procedure could introduce selection bias.

**Response:** We appreciate the Reviewer’s comment on this point, according to which we changed the following sentences in the Methods section from

“Subjects were randomly assigned by attending physicians to the MI-E or control group with a 1:1 ratio using software (FileMaker pro 16; FileMaker, Inc., Santa Clara, CA, USA). The randomization process did not include random blocking.”

to

“Subjects were randomly assigned by attending physicians to the MI-E or control group with a 1:1 ratio. Included subjects were assigned a random number of 0 or 1 using software (FileMaker pro 16; FileMaker, Inc., Santa Clara, CA, USA), designating them as members of the control group or MI-E group, respectively. The randomization process did not include random blocking.” (line 114-118)

**Comment 11:** Could the authors clarify the intervention with regard to indication for use and frequency of use per day? Currently it could be that one patient received the MI-E every hour and another only a few times a day.

**Response:** We wish to express our deep appreciation to the Reviewer for the insightful comment on this point. More precise details were needed on the frequency and method of suction. Suctioning was performed on-demand. Although subjects who required suctioning at least once every hour at the start of the study were included in the research, there was no upper limit to the number of times. Moreover, if it was not possible to suction airway secretions after inclusion, and no further suctioning of airway secretions was expected to be possible based on a physical examination and auscultation, suctioning was allowed to occur less frequently than once an hour. This may have affected the results obtained. However, performing additional suctioning on patients who do not produce further airway secretions despite suctioning may result in more side effects than benefits; therefore, we were unable to maintain the same frequency for all patients. We documented this as a limitation in the Discussion section.

In accordance with the Reviewer’s comment, we changed the following sentences from

“However, if they no longer needed to suction frequently based on the clinical assessment after study inclusion, they were allowed to suction less often than once every hour.”

to

“Suctioning was performed on-demand by the nurse in charge who fully understood the protocol of the study and had received adequate training and practical experience with suctioning. Although subjects who required suctioning at least once every hour at the start of the study were included in the research, there was no upper limit to the number of times. Moreover, if it was not possible to suction airway secretions after inclusion and no further suctioning of airway secretions was expected to be possible based on a physical examination and auscultation, suctioning was allowed to occur less frequently than once an hour.” (line 131-139)

and

“In the MI-E group, if there were no more airway secretions to be cleared even after using MI-E, suctioning and the use of MI-E were permitted at intervals exceeding one hour.” (line 146-148)

Furthermore, as suggested, one patient may have received MI-E every hour and another only a few times a day. Therefore, the following sentences were added to the Discussion section.

“It was difficult to establish a fixed number of suctions per hour because additional suctioning or the use of MI-E in the absence of further cleared airway secretions may have led to potential side effects without corresponding benefits. However, variability in the frequency of suctioning and MI-E sessions that individual patients received each day may have affected the outcomes.” (line 334-339)

**Comment 12:** Additionally it is unclear how patients were humidified during mechanical ventilation and what other airway care interventions were performed, for example nebulization therapy or manual hyperinflation. In the current description there is a risk of performance bias.

**Response:** We appreciate the Reviewer’s comment on this point, according to which the following sentence on humidification methods and the use of nebulizers was added to the Methods section.

“Humidification was performed by connecting a heated humidifier to the continuous tube from the endotracheal tube. When the nurse considered the use of a nebulizer to be more effective at facilitating the expectoration of airway secretions, the nebulizer was used on-demand.” (line 149-152)

Since the decision regarding when to use the nebulizer needed to be more clearly defined in the study protocol and the absence of clear criteria in this research may have led to the possibility of selection bias, the following sentences were added to the Discussion section.

“The timings of suctioning and nebulization were selected by nurses based on a comprehensive assessment, including physical and auscultation findings and whether airway secretions had been cleared during the previous suctioning. However, due to the absence of standardized rules for the timing of suctioning and nebulization, there was a potential for selection bias.” (line 314-318)

**Comment 13:** Could the authors describe what they defined as adverse events in this study?

**Response:** We wish to express our deep appreciation to the Reviewer for the insightful comment on this point. We agree that a clearer definition of adverse events was needed before commencing the study. Adverse events were not defined before and not taken systematically in the present study. Adverse events were noted in a retrospective review of medical records. In 13 previous studies using MI-E, 10 found no adverse events, while 3 described oxygen desaturation, hemodynamic variation, pneumothorax, mucus plugging, hemoptysis, and chest pain as adverse events. (Swingwood E, Stilma W, Tume LN, Cramp F, Voss S, Bewley J, et al. The Use of Mechanical Insufflation-Exsufflation in Invasively Ventilated Critically Ill Adults Respir Care. 2022 ;67(8):1043-1057.) Based on adverse events in previous studies, a retrospective review did not reveal any adverse events. The following detailed description on adverse events was added to the Results section.

“While some patients had hemodynamic instability or arrhythmia at the time of inclusion, no new cases of hemodynamic instability or arrhythmia occurred with the use of MI-E. The use of MI-E did not lead to new cases of lung injury due to barotrauma, volutrauma, atelectrauma, or biotrauma. No other apparent adverse effects attributed to MI-E occurred in the present study, such as pneumothorax, hemoptysis, oxygen desaturation, and mucus plugging.” (line 232-237)

**Comment 14:** Reference include many articles with regard to patient with neuromuscular disease. In the last year more studies have been published with regard to MI-E in invasively ventilated patients. Please have another reading of the literature and integrate these in the discussion. For example the Scoping review with an overview of adverse events in invasively ventilated patients (PMID: 35610033) and the study of Marti (PMID: 36041752) and Wu (PMID:36041752).

**Response:** We wish to express our deep appreciation to the Reviewer for the insightful comment on this point. The following sentences on recent studies were added to the Discussion section.

“Although a previous study that investigated the use of MI-E in healthy Landrace-Large White female pigs showed that +40/-70 cmH_2_O was the most effective pressure combination [16], MI-E settings with a pressure of ±40 cmH_2_O are most commonly used in invasively ventilated critically ill patients. [17]” (line 293-296)

and

“In 13 studies that used MI-E, 10 found no adverse events, while 3 reported oxygen desaturation, hemodynamic variation, pneumothorax, mucus plugging, hemoptysis, and chest pain as adverse events. [17]” (line 302-304)

and

“A previous study suggested that the use of MI-E increased the incidence of chest pain. [25]” (line 301-302)

**Comment 15:** The Kaplan Meyer is nice to add, but could be made more clear by a distinction between the groups. Maybe a continuous line and dots or stripes would be more clear for reading?

**Response:** We appreciate the Reviewer’s comment on this point, according to which the line for the control group in Figure 2 was changed to a dashed line to make it more distinguishable.

Thank you again for your comments. We trust that the revised manuscript will now be considered suitable for publication.
