# Supplementary material for: Effects of mechanical insufflation-exsufflation on ventilator-free days in intensive care unit subjects with sputum retention; a randomized clinical trial
Source: PLoS One. 2024 May 2;19(5):e0302239. doi: 10.1371/journal.pone.0302239 (PMC11065296; doi:10.1371/journal.pone.0302239)
Supplement: Attachment — Submitted filename: Response to Reviewer.docx [file pone.0302239.s010.docx]

Dear Dr. Lascarrou,

Thank you for your e-mail regarding our manuscript entitled “Effects of mechanical insufflation-exsufflation on ventilator-free days in intensive care unit subjects with sputum retention; A randomized clinical trial”, and the valuable comments of the Reviewer. Please find attached our revised manuscript as well as point-by-point responses to the Reviewer’s comments.

As indicated by the Reviewer, there was an error in one of the references. We sincerely apologize for the incorrect citation. We changed the reference in the sentence “Since another study demonstrated that pressures of ±54 cmH_2_O were tolerated well” (line 306-307) from

“Bach JR, Mehta AD. Respiratory muscle aids to avert respiratory failure and tracheostomy: a new patient management paradigm. Journal of Neurorestoratology. 2014;2:25-35.”

to

“Bach JR, Sinquee DM, Saporito LR, Botticello AL. Efficacy of mechanical insufflation-exsufflation in extubating unweanable subjects with restrictive pulmonary disorders. Respir Care. 2015 Apr;60(4):477-83.”

We consider the revised manuscript to be a suitable response to the Reviewer’s comments and it has been significantly improved over the previous submission. We trust that it is now suitable for publication in *PLOS ONE*. Thank you in advance for your kind consideration of our manuscript.

Sincerely yours,

Shota Kubota

Department of Emergency and Critical Care Medicine, Hitachi General Hospital, Ibaraki, Japan

**Comment 1.** The exclusion criteria do not contain pneumothorax, which is the main adverse event when using MI-E.

Previous Response: We appreciate the Reviewer’s comment on this point. Patients who were considered to have underlying conditions that may be exacerbated by MI-E were excluded by the attending physician. The following sentence in the Methods section was changed from

“Patients who met the following criteria were excluded from the study: pregnant women, […], and cases that were deemed inappropriate by the attending physician.”

to

“Patients who met the following criteria were excluded from the study: pregnant women, […], and patients considered by the attending physician to have a coexisting condition that is exacerbated by MI-E, such as pneumothorax, empyema with fistula, or hemoptysis.” (line 96-101)

Comment: I accepted the authors' response .However, quality-assured clinical studies should include details of exclusion criteria in the protocol.

**Response:** We wish to thank the Reviewer for this comment, with which we agree.

We apologize for not providing a detailed definition of exclusion criteria in the study protocol. The following sentences were added to the Discussion section.

“Regarding the exclusion criteria of the present study, patients considered by the attending physician to have a coexisting condition exacerbated by MI-E, such as pneumothorax, empyema with fistula, or hemoptysis, were excluded. However, since the study protocol did not provide a detailed definition of exclusion criteria, there was a potential for selection bias.” (line 353-357)

**Comment 2.** More than 80% of the subjects are male, but how many have a smoking history or chest C T that may have a bullae? The MI-E pressure of 40 cm H2O was performed in all the same cases, but it is necessary to mention how it was managed and whether there were no adverse events.

Previous Response: We appreciate the Reviewer’s comment on this point. In the present study, the collection of smoking history and chest CT scans were not specified in the study protocol. However, at Hitachi General Hospital, routine smoking history inquiries are conducted upon admission. Therefore, we retrospectively examined the smoking history of all patients. Additionally, since all patients included in the present study had undergone chest CT imaging, we retrospectively assessed the percentage of patients with lung bullae. The results obtained showed that 33% of patients in the control group and 48% in the MI-E group had a history of smoking, while 62% in the control group and 59% in the MI-E group had bullae in the lungs.

We also provided more detailed information on how to connect the MI-E machine and adjust pressure settings in the Methods section.

“When the MI-E COMFORT COUGH-II is directly connected to the endotracheal tube and the pressure is set to 40 cmH_2_O, it applies both positive and negative pressures of 40 cmH_2_O during inhalation and exhalation, respectively, inside the endotracheal tube.” (line 141-144)

More detailed descriptions of actual adverse events were also added to the Results section.

“While some patients had hemodynamic instability or arrhythmia at the time of inclusion, no new cases of hemodynamic instability or arrhythmia occurred with the use of MI-E. Furthermore, the use of MI-E did not lead to new cases of lung injury due to barotrauma, volutrauma, atelectrauma, or biotrauma. No other apparent adverse effects attributed to MI-E occurred in the present study, such as pneumothorax, hemoptysis, oxygen desaturation, and mucus plugging.” (line 232-237)

Based on previous studies, the following sentences on the adverse event of pneumothorax, in connection with a smoking history or bullae were added to the Discussion section.

“In 13 studies that used MI-E, 10 found no adverse events, while 3 reported oxygen desaturation, hemodynamic variation, pneumothorax, mucus plugging, hemoptysis, and chest pain as adverse events. In the present study, 33% of patients in the control group and 48% in the MI-E group had a history of smoking. All patients had undergone chest CT, and the percentage of patients with bullae in the lungs was 62% in the control group and 59% in the MI-E group. Pneumothorax did not occur in patients, even in those with a history of smoking or bullae.” (line 302-309)

Comment: I understood authors response. If the author mentions a history of smoking or bullae in the discussion, it should be described in the results.

In the MI-E group, 59% of patients had pulmonary bullae but did not experience any adverse events. This is an important result for the risk of MI-E.

The maximum size of the bullae is an important information with regard to the risk of MI-E.

**Response:** We strongly appreciate the Reviewer’s comment on this point. We added the percentage of patients with a history of smoking and bullae, and the maximum size of bullae to Table 1 in the Results section. We also revised the description on adverse events in the Discussion section with the following text.

“In the present study, 33% of patients in the control group and 48% in the MI-E group had a history of smoking. All patients had undergone chest CT, and the percentage of patients with bullae in the lungs was 62% in the control group and 59% in the MI-E group. The median of the maximum size of bullae was 12 mm (IQR, 5-17) in the control group and 12 mm (IQR, 7-30) in the MI-E group. Pneumothorax did not occur in patients, even in those with a history of smoking or bullae.” (line 311-318)

**Comment 3.** Reference (21) needs to be checked, as it does not mention the sentence "since another study showed that a pressure of ±54 cmH2O is well tolerated". The reference to be cited is "DOI:https://doi.org/10.4187/respcare.03584", which has been previously noted.

**Response:** We appreciate the Reviewer’s comment on this point, with which we agree. We sincerely apologize for the incorrect citation of references. We changed the reference in the sentence “Since another study demonstrated that pressures of ±54 cmH_2_O were tolerated well” (line 306-307) from

“Bach JR, Mehta AD. Respiratory muscle aids to avert respiratory failure and tracheostomy: a new patient management paradigm. Journal of Neurorestoratology. 2014;2:25-35.”

to

“Bach JR, Sinquee DM, Saporito LR, Botticello AL. Efficacy of mechanical insufflation-exsufflation in extubating unweanable subjects with restrictive pulmonary disorders. Respir Care. 2015 Apr;60(4):477-83.”

**Comment 4:** Smoking is known to increase the amount of airway mucus. It may be mentioned in the discussion that smoking history was higher in the MI-E group (48%) than in the control group (33%) may have affected the results.

In a discussion, the description needs to be more structured for ease of reading.

1. Summary of research hypotheses and results

2. Discussion of the efficacy of MI-E

3. Consideration of adverse events.

4. Limitations

In this manus, I think the above structure is used.

In the discussion of efficacy in 2,

Firstly, the sample size that is considered to have the most effect. The next structure is to describe the influence of smoking history, medical factors and MI-E pressure settings.

This structure would be easier for the reader to understand.

**Response:** We wish to express our deep appreciation to the Reviewer for their insightful comment on this point. We added the following text to 2 ("Reasons for the absence of significant findings") to make it easier for the reader to understand.

“Several factors may account for the observed lack of a significant increase in the number of ventilator-free days with MI-E. The primary contributing factor is the limited sample size. Prior to the initiation of the present study, the target difference in ventilator-free days between the two groups was designed as 4 days and the sample size was set at 60 subjects. We planned to terminate enrollment once the target sample size was reached or in June 2019. Fewer ICU-ventilated subjects met the inclusion criteria than expected, and, thus, the study was terminated before the target sample size was reached. The insufficient sample size may have affected the power of the study. Therefore, randomized open-label trials with larger sample sizes are needed. Furthermore, the percentage of patients with a history of smoking may have affected the results. In the present study, 33% of patients in the control group and 48% in the MI-E group had a history of smoking. Since smoking is known to increase the amount of airway mucus [13], the higher percentage of patients with a smoking history in the MI-E group than in the control group may also have affected the results. In addition, medical factors may have contributed to the lack of a significant difference. In patients without neuromuscular disease, even if airway secretions are high, the ability to exhale may be sufficient without MI-E. While the use of MI-E may reduce airway resistance or enhance compliance [7,8], it does not directly address the underlying medical factors (such as pneumonia, cardiac arrest, sepsis, or surgery) that necessitated intubation. This may explain why the number of ventilator-free days was not significantly different. Finally, the possibility that the pressure settings for MI-E were inappropriate may be a reason for the lack of a significant difference. A pressure of ±40 cmH_2_O may have been insufficient for patients with excessive sputum production, such as those examined in the present study. On the other hand, although MI-E was effective, potential adverse events, such as barotrauma or atelectrauma, may have affected the results obtained.” (line 254-278)

I hope that the modified structure, along with the additional sentences, has enhanced readability for the readers.

**Comment 5:** The order of the MI-E and control groups in Tables 1 and 2 is not aligned. They should be aligned in order to show the reader clearly.

**Response:** We wish to thank the Reviewer for this comment. We reversed the order of the control group and MI-E group in Table 2 to match that in Table 1.

We wish to thank the Reviewer again for their valuable comments.
