# Supplementary material for: Effects of mechanical insufflation-exsufflation on ventilator-free days in intensive care unit subjects with sputum retention; a randomized clinical trial
Source: PLoS One. 2024 May 2;19(5):e0302239. doi: 10.1371/journal.pone.0302239 (PMC11065296; doi:10.1371/journal.pone.0302239)
Supplement: Attachment — Submitted filename: Response to Reviewers.docx [file pone.0302239.s011.docx]

Dear Dr. Lascarrou,

Thank you for your e-mail regarding our manuscript entitled “Effects of mechanical insufflation-exsufflation on ventilator-free days in intensive care unit subjects with sputum retention; A randomized clinical trial”, and the valuable comments of the Reviewer. Please find attached our revised manuscript as well as point-by-point responses to the Reviewer’s comments. We consider the revised manuscript to be a suitable response to the Reviewer’s comments and it has been significantly improved over the previous submission. We trust that it is now suitable for publication in PLOS ONE. Thank you in advance for your kind consideration of our manuscript.

Sincerely yours,

Shota Kubota

Department of Emergency and Critical Care Medicine, Hitachi General Hospital, Ibaraki, Japan

We wish to express our strong appreciation to the Reviewer for their insightful comments, which have helped us to significantly improve the quality of the revised manuscript.

**Comment 1.** For randomisation; no block used, therefore assumption is that, this was a simple randomisation - authors include this in the manuscript.

**Response:** We wish to thank the Reviewer for this comment. The following sentence in the Methods section was changed from

“The randomization process did not include random blocking.”

to

“Simple randomization was implemented and did not include random blocking.” (lines 117-118)

The following sentence in the Discussion section was changed from

“The number of patients in both groups was not 1:1, which may have affected the results obtained because the randomization process did not include random blocking.”

to

“The number of patients in both groups was not 1:1, which may have affected the results obtained because simple randomization was implemented and did not include random blocking.” (lines 361-363)

**Comment 2.** This was an open-label study, when was the analysis plan finalized, ideally prior to first participant recruited.

**Response:** We appreciate the Reviewer’s comment on this point. The following sentence was added to the Methods section.

“The analysis plan was finalized on 16th October 2017, prior to the recruitment of the first participant on 1st November 2017.” (lines 189-191)

**Comment 3.** In statistical analysis section, mention how missing data would be handled if at all?

**Response:** We thank the Reviewer for this comment. The following sentence was added to the Results section.

“There were no missing data.” (line 209)

**Comment 4.** Define your population of analysis (e.g analysed as randomised irrespective of whether they received the intervention or not).

**Response:** We appreciate the Reviewer’s comment on this point. The following sentence was added to the Methods section.

“In the present study, we performed an intention-to-treat analysis.” (line 191)

**Comment 5.** Table 1- not recommended to test for baseline characteristics as this an RCT, any differences observed would be due to chance - omit p-values from table 1 as mis-leading.

**Response:** We strongly appreciate the Reviewer’s comment on this point. We omitted p-values from table 1.

**Comment 6.** Such a wide gap of age, it would help if you can present the age-category into sensible categories, to understand the distribution of who is included in your sample?

**Response:** We wish to express our deep appreciation to the Reviewer for the insightful comment on this point. We added age categories to Table 1. Patients in the Control group and MI-E group were categorized into three age groups: 60 years and younger, between 61 and 80 years, and 81 years and older.

**Comment 7.** For descriptions purposes, also include a sensible cut-off of the primary outcome, i.e curious that you have less days in the control group - hence why possible interest in age categories, i.e could be because they may be more younger people in control group that intervention group - influencing ventilator free days.

**Response:** We strongly appreciate the Reviewer’s comment on this point. Since the difference in age distribution among patients in the Control group and MI-E group may have potentially affected the results obtained, the following sentence was added to the Discussion section.

“Patients in the control group were younger than those in the MI-E group, which may have affected the results obtained.” (lines 269-271)

Based on the present results on the duration of mechanical ventilation, the reduction of four days for sample size estimations may have been too substantial. The following sentences were added to the Discussion section.

“In the present study, the sample size was calculated to detect a reduction of 4 ventilator-free days in the MI-E group for the primary endpoint. Since the present results showed that the duration of mechanical ventilation was 6 in the MI-E group and 8 in the control group, setting the cut-off of the primary outcome at 4 days may have been too large.” (lines 363-367)

We wish to thank the Reviewer again for their valuable comments.
